# Type 2 Diabetes and Financial Outcomes

**DOI:** 10.1001/jamanetworkopen.2025.23453

**Published:** 2025-07-28

**Authors:** Matthew Pesavento, Cäzilia Loibl, Stephanie Moulton, Donald Haurin, Madison Hyer, Djhenne Dalmacy, Joshua J. Joseph

**Affiliations:** 1 The Ohio State University, Columbus

## Abstract

**Importance:**

Type 2 diabetes is common and associated with negative financial health.

**Objective:**

To investigate the association of type 2 diabetes with adverse financial outcomes and identify patient groups at risk.

**Design, Setting, and Participants:**

This economic evaluation study analyzed electronic health records linked to credit records of adults aged 18 years or older with at least 1 medical encounter at a primary care medical center in Ohio from October 1, 2017, to December 31, 2021. Patient health records were included if they reported a glycated hemoglobin value, diagnostic code for type 2 diabetes, or a prescription for antidiabetic medications.

**Exposure:**

Type 2 diabetes.

**Main Outcome and Measures:**

The main outcome was adjusted probability of any adverse financial outcomes, below-prime credit score, nonmedical and medical debt in collections, delinquent debt, debt charge-offs, bankruptcy filing, and foreclosure. Adjustments included age, sex, race, Hispanic ethnicity, wage earnings, health insurance type, exposure period, and quarter-year indicators.

**Results:**

The study population included 166 285 patients (mean [SD] age, 52.3 [15.3] years; 55.0% female; 0.2% of American Indian or Alaska Native or Native Hawaiian or Pacific Islander, 3.5% Asian, 19.1% Black, and 73.2% White race; 0.8% identifying as multiracial; 2.1% of Hispanic and 97.9% of non-Hispanic ethnicity), of whom 41.7% were diagnosed with type 2 diabetes. In the analytic sample, 50.8% lacked earned income, and 32.6% had Medicare coverage. For patients with vs without diabetes, estimated probabilities were significantly higher for any adverse financial outcomes (64.5% [95% CI, 64.1%-64.9%] vs 49.9% [95% CI, 49.6%-50.2%]), below-prime credit score (59.7% [95% CI, 59.3%-60.1%] vs 45.9% [95% CI, 45.6%-46.2%]), medical collections (36.9% [95% CI, 36.5%-37.3%] vs 23.9% [95% CI, 23.7%-24.2%]), nonmedical collections (38.4% [95% CI, 38.0%-38.8%] vs 27.7% [95% CI, 27.5%-28.0%]), delinquent debt (23.3% [95% CI, 22.9%-23.7%] vs 15.6% [95% CI, 15.4%-15.8%]), debt charge-offs (15.4% [95% CI, 15.1%-15.8%] vs 10.1% [95% CI, 9.9%-10.2%]), bankruptcy filings (2.1% [95% CI, 2.0%-2.3%] vs 1.4% [95% CI, 1.3%-1.5%]), and foreclosures (0.5% [95% CI, 0.5%-0.6%] vs 0.3% [95% CI, 0.2%-0.4%]). Patients with diabetes experienced more adverse financial outcomes (1.9 vs 1.2) and had lower credit scores (mean [SE], 618.7 [0.4] vs 664.2 [0.5]) than those without diabetes. For patients with vs without diabetes, the maximum amount of nonmedical debt in collection was higher ($1875 [95% CI, $1834-$1916] vs $1361 [95% CI, $1333-$1389]), as was delinquent debt ($11 387 [95% CI, $10 796-$11 977] vs $7630 [95% CI, $7305-$7955]). The adjusted prevalence of adverse financial outcomes was higher among patients of Black race, enrolled in Medicaid, of Hispanic ethnicity, younger than 65 years, without earned income, and of female sex.

**Conclusions and Relevance:**

This economic evaluation of patient health records linked to credit records and wage earnings shows a positive association of type 2 diabetes with adverse financial outcomes. These findings suggest that patients with type 2 diabetes may experience substantially more adverse financial outcomes compared with patients without diabetes, highlighting the need to consider patient financial health when treating type 2 diabetes, particularly for patient groups at higher risk.

## Key Points

**Question:**

What is the association of type 2 diabetes with adverse financial outcomes among adult patients of a Midwestern medical center, and which patients are at greater risk?

**Findings:**

In this economic evaluation study of 166 285 adult patients, the probability of adverse financial outcomes was significantly higher among patients with type 2 diabetes, with higher risk observed among patients of Black race, enrolled in Medicaid, of Hispanic ethnicity, younger than 65 years, without earned income, and of female sex.

**Meaning:**

These findings suggest that patients with type 2 diabetes may experience substantially more adverse financial outcomes compared with patients without diabetes, highlighting the need to consider patient financial health when treating type 2 diabetes, particularly for patient groups at higher risk.

## Teaser

This economic evaluation study assesses the association of type 2 diabetes with adverse financial outcomes and investigates which patient groups are at greater risk.

## Introduction

The association of diabetes and financial stress has been well documented. Approximately 20% to 25% of adults (aged ≥18 years) with diabetes have been reported to ration insulin, and approximately 30% have rationed diabetes supplies.^1,2,3^ Medication nonadherence has been linked to a higher risk of foregone or delayed medical care, food insecurity, hospitalizations, and mortality.^4,5^

While the financial burdens for individuals with diabetes are well documented,^6,7,8^ limited information is available on how these burdens are associated with financial outcomes. A study of commercially insured adults in Michigan found that an estimated 5% to 10% of working adults with diabetes have medical debt in collection of close to $100.^9^ More detailed analysis is needed to examine other financial outcomes associated with diabetes. Our objective was to assess the association of type 2 diabetes with financial outcomes using a new dataset that links patient electronic health records (EHRs) to commercial credit data and government wage data for a 4.25-year period for patients at a large medical center in Central Ohio. Specifically, this study examines the association of prevalent type 2 diabetes with below-prime credit scores, medical and other debt in collections, delinquent debt, debt charge-offs, bankruptcy filing, and foreclosure.

## Methods

### Data Sources and Study Population

This economic evaluation study used EHR-derived data of The Ohio State University Wexner Medical Center patients with a glycated hemoglobin (HbA_1c_) measure, an *International Statistical Classification of Diseases, Tenth Revision* code for type 2 diabetes, or an antidiabetic medication prescribed during a medical encounter between October 1, 2017, and December 31, 2021. The study was approved by The Ohio State University Institutional Review Board, which waived informed consent due to the use of secondary, deidentified data. This study followed the Consolidated Health Economic Evaluation Reporting Standards (CHEERS) reporting guideline.^10^

The EHR variables were extracted at the patient-encounter level between March 1 and June 30, 2022, and aggregated by quarter (N = 212 177). We excluded from the sample patients with type 1 diabetes (n = 6863), patients ever pregnant during the study period (n = 443), patients without valid personal identifiers (ie, Social Security number) (n = 26 812), and patients with incident type 2 diabetes (n = 6405). Listwise deletion of missing values (n = 5369) resulted in an analytic sample of 166 285 patients in treatment and comparison groups (eFigure in Supplement 1).

Credit data were from a major US credit bureau that compiles administrative financial data from lenders and creditors for approximately 95% of the Ohio adult population. The individual-level dataset lists credit types, account balances, overdue payments, loans charged-off and sent to collections, bankruptcies, and foreclosures.^11^ We used quarter-end balances for this study. Employment data were from the Ohio Longitudinal Data Archive, which includes all workers with employment earnings reported to the state of Ohio. The Center for Human Resource Research, a National Institute of Standards and Technology 800-53 secure data center with Health Insurance Portability and Accountability Act compliance, linked the EHR, credit, and employment data with a secure hashing process based on patients’ Social Security numbers.

Identification criteria for type 2 diabetes included an HbA_1c_ of 6.5% or higher (to convert to proportion of total hemoglobin, multiply by 0.01); antidiabetic medication prescriptions; or an *International Statistical Classification of Diseases, Tenth Revision* diagnosis code for type 2 diabetes in the patient’s problem list, medical history, or billing diagnosis during the study period. The comparison sample included patients who did not have any of the 3 indicators in their problem list, medical history, or billing diagnosis during the study period (eTable 1 in Supplement 1).

### Outcome Measures, Independent Variables, and Covariates

Outcomes were constructed from credit data, including a low credit score (≤660), medical and nonmedical debt in collections, 60-plus–day delinquent debt, debt charge-offs, bankruptcy filing, and foreclosure (eMethods in Supplement 1).^9,12^ Credit score was from VantageScore 4.0 (VantageScore Solutions), ranging from 300 to 850, reflecting the consumer’s overall credit history. We used a continuous measure and constructed a binary indicator for credit scores at or below 660, which is classified by industry as below-prime, higher-risk borrowers.^13^

Medical and nonmedical debt in collections was debt held at a collection agency. The 60-plus–day delinquent debt included 60- to 180-day late, unpaid financial obligations not charged off. Charged-off debt included severely delinquent debt transferred to a collection agency or debt buyer. Bankruptcy included Chapter 7 or 13 bankruptcy filings in a quarter. Foreclosure indicated that a first mortgage was reported as being foreclosed by the creditor. Debt in collections, delinquent debt, charged-off debt, bankruptcy filing, and foreclosure were coded as a binary measure of ever having had such outcomes during a given quarter (coded as 1 or as 0 if otherwise) and as the maximum amount of debt in collections and delinquent debt at the end of a quarter (highest quarterly balance). Summative outcomes included a binary measure of patients experiencing any of the 7 adverse financial outcomes, as well the count of adverse financial outcomes (0-7).

Covariates from the EHR included age (18-34, 35-49, 50-64 [reference group], ≥65 years), sex (male = 0, female = 1), race (American Indian or Alaska Native or Native Hawaiian or Pacific Islander [grouped due to low sample numbers], Asian, Black, White [reference group], multiracial, missing), and Hispanic ethnicity (yes = 1, no = 0). Race and ethnicity were included because they are known risk factors of type 2 diabetes. Covariates from wage data included a binary indicator for having any reported wage earnings as of the baseline period (October to December 2017) and the amount of quarterly wage earnings reported, in categories ($1-$9999, $10 000-$19 999 [reference group], $20 000-$29 999, $30 000-$39 999, ≥$40 000). We interacted the binary indicator for no wages as of the first quarter (Q1) with the age category of 65 years or older, as no wage earnings may have a different association with adverse financial outcomes after older adults exit the labor force compared with younger adults. Covariates from credit data included the duration of the exposure period (1-17 quarters) and 16 binary quarter-year indicators for the outcome measure (outcome observed = 1, outcome not observed = 0) to account for changes in policy or macroeconomic conditions over time (Q4 2017 to Q4 2021 [reference category]). Covariates from EHR billing data included ever having a given type of health insurance (private, Medicare, Medicaid, other [Ohio Bureau of Workers Compensation and US Veterans Administration, as well as coverage through the Bureau of Disability, Columbus Free Clinic, drug advocacy programs, and programs supporting people harmed by criminal acts], self-pay) recorded during the exposure period.

### Statistical Analysis

We measured adverse financial outcomes during the quarters for which we had evidence that a patient had a type 2 diabetes diagnosis or not (exposure period). For patients with type 2 diabetes, the exposure period began with the first calendar-quarter of an EHR with evidence of a diabetes diagnosis through the end of the study period (Q4 2021). For comparison group patients, the exposure period began with the beginning of the study period (Q4 2017) through their final observation in the EHR.

We estimated the association of type 2 diabetes with adverse financial outcomes ever occurring during the patient’s exposure period using generalized linear model regression (binomial family) and logit link function. For the count of adverse financial outcomes, we used negative binomial regression models with a log link. For continuous dollar amount of debts (collections, delinquencies), we used 2-part exponential hurdle models to simultaneously estimate the adjusted probability of the patient experiencing a given type of debt and the conditional maximum amount of debt during their exposure period (highest quarterly balance). We standardized credit scores by subtracting the lowest score of the study period from the top score. We fit a generalized linear model regression for this continuous γ-distributed response (0-550) and log link function. All specifications were controlled for age, sex, race, Hispanic ethnicity, wage earnings, health insurance type, exposure period in quarters, and quarter-year indicators. To facilitate interpretation, we calculated the marginal effect of type 2 diabetes on the given outcomes and reported the difference of the marginal effects for patients with and without diabetes. The threshold for significance was set at a 2-sided *P* < .05. The data analyses were performed using Stata/MP, version 18.5 (StataCorp LLC).

## Results

The analytic sample included 166 285 patients (mean [SD] age, 52.3 [15.3] years; 55.0% female and 45.0% male; 0.2% of American Indian or Alaska Native or Native Hawaiian or Pacific Islander, 3.5% of Asian, 19.1% of Black, and 73.2% of White race; 0.8% identifying as multiracial; 2.1% of Hispanic and 97.9% of non-Hispanic ethnicity) (Table 1). A total of 69 371 patients (41.7%) had a type 2 diabetes diagnosis. In the analytic sample, 50.8% lacked earned income, and 32.6% had Medicare coverage. Patients with vs without diabetes were older (mean [SD] age, 59.8 [14.2] vs 47.0 [16.1] years), less often female (50.8% vs 58.0%) and of Hispanic ethnicity (1.8% vs 2.2%), but more often of Black race (20.7% vs 17.9%). There was no statistically significant difference in the share of White patients with and without diabetes. Patients with diabetes were more often without wage earnings at baseline (65.1% vs 40.6%), and among those with wage earnings at baseline, the earnings were lower (mean [SD], $11 477 [$21 796] vs $15 400 [$42 359]). The mean (SD) exposure period for patients with diabetes was 12.9 (5.1) quarters and for the comparison group without diabetes, 14.2 (4.1) quarters.

**Table 1.** Sample Demographic Characteristics

Characteristic	No. (%)			SMD
	Total sample (N = 166 285)	Patients with type 2 diabetes diagnosis (n = 69 371)	Patients without type 2 diabetes diagnosis (n = 96 914)	
Diabetes diagnosis	69 371 (41.7)	69 371 (100)	0	NA
Age (Q4 2017), y				
Mean (SD)	52.3 (15.3)	59.8 (14.2)^a^	47.0 (16.1)	−0.8
18-34	30 351 (18.3)	4310 (6.2)^a^	26 041 (26.9)	0.6
35-49	41 231 (24.8)	12 637 (18.2)^a^	28 594 (29.5)	0.3
50-64	54 688 (32.9)	26 883 (38.8)^a^	27 805 (28.7)	−0.2
≥65	40 015 (24.1)	25 541 (36.8)^a^	14 474 (14.9)	−0.5
Sex				
Female	91 463 (55.0)	35 229 (50.8)^a^	56 234 (58.0)	0.1
Male	74 822 (45.0)	34 142 (49.2)^a^	40 680 (42.0)	−0.1
Race				
American Indian or Alaska Native or Native Hawaiian or Pacific Islander	396 (0.2)	169 (0.2)	227 (0.2)	0.0
Asian	5787 (3.5)	1623 (2.3)^a^	4174 (4.3)	0.1
Black	31 720 (19.1)	14 347 (20.7)^a^	17 373 (17.9)	−0.1
White	121 679 (73.2)	50 649 (73.0)	71 030 (73.3)	0.0
Multiracial	1292 (0.8)	381 (0.5)^a^	911 (0.9)	0.0
Missing	5401 (3.2)	2202 (3.2)	3199 (3.3)	0.0
Hispanic ethnicity				
Yes	3438 (2.1)	1264 (1.8)^a^	2174 (2.2)	0.0
No	162 847 (97.9)	68 107 (98.2)^a^	94 740 (97.8)	0.0
Wage earnings (Q4 2017), $				
Mean (SD)^b^	14 239 (37 468)	11 477 (21 796)^a^	15 400 (42 359)	0.1
0	84 555 (50.8)	45 182 (65.1)^a^	39 373 (40.6)	−0.5
1-9999	39 426 (23.7)	13 751 (19.8)^a^	25 675 (26.5)	0.2
10 000-19 999	26 730 (16.1)	7248 (10.4)^a^	19 482 (20.1)	0.3
20 000-29 999	10 223 (6.1)	2252 (3.2)^a^	7971 (8.2)	0.2
30 000-39 999	3084 (1.9)	602 (0.9)^a^	2482 (2.6)	0.1
≥40 000	2267 (1.4)	336 (0.5)^a^	1931 (2.0)	0.1
Health insurance type^c^				
Private	86 496 (52.0)	22 674 (32.7)^a^	63 822 (65.9)	0.7
Medicare	54 185 (32.6)	32 137 (46.3)^a^	22 048 (22.8)	−0.5
Medicaid	30 772 (18.5)	12 498 (18.0)^a^	18 274 (18.9)	0.0
Other^d^	6489 (3.9)	2519 (3.6)^a^	3970 (4.1)	0.0
Self-pay	10 885 (6.5)	3707 (5.3)^a^	7178 (7.4)	0.1

Abbreviations: NA, not applicable; Q4, quarter 4; SMD, standardized mean difference.

^a^ *P* < .001.

^b^ Among those with any wage earnings.

^c^ Ever in study period.

^d^ Other insurance included Ohio Bureau of Workers Compensation and US Veterans Administration, as well as coverage through the Bureau of Disability, Columbus Free Clinic, drug advocacy programs, and programs supporting people harmed by criminal acts.

During the study period, 56.1% of the sample had any adverse financial outcomes, with 51.7% having below-prime credit scores, 32.2% with debt in nonmedical collections, 29.2% with debt in medical collections, 18.5% with debt overdue, 12.1% with debt charge-offs, 1.7% filing bankruptcy, and 0.4% experiencing foreclosure (Table 2). Patients with vs without diabetes had greater exposure to adverse financial outcomes (63.5% vs 50.7%); had below-prime credit scores (58.5% vs 46.9 %), nonmedical collections (37.9% vs 28.2%), medical collections (35.3% vs 24.9%), debt overdue (20.4% vs 17.2%), and debt charge-offs (13.9% vs 10.8%); filed bankruptcy (1.8% vs 1.6%); and had been foreclosed (0.5% vs 0.3%).

**Table 2.** Financial Outcome Measures

Characteristic	No. (%)			SMD
	Total sample (N = 166 285)	Patients with type 2 diabetes diagnosis (n = 69 371)	Patients without type 2 diabetes diagnosis (n = 96 914)	
**Credit across study period** ^a^				
Ever any adverse financial outcomes (past 90 d)	93 208 (56.1)	44 057 (63.5)^b^	49 151 (50.7)	−0.3
Ever Vantage credit score ≤660 (as of end of quarter)	85 992 (51.7)	40 570 (58.5)^b^	45 422 (46.9)	−0.2
Ever any nonmedical debt in collections (as of end of quarter)	53 575 (32.2)	26 290 (37.9)^b^	27 285 (28.2)	−0.2
Ever any medical debt in collections (as of end of quarter)	48 591 (29.2)	24 478 (35.3)^b^	24 113 (24.9)	−0.2
Ever any 60-plus–day delinquent debt (as of end of quarter)	30 742 (18.5)	14 120 (20.4)^b^	16 622 (17.2)	−0.1
Ever any charge-offs (past 90 d)	20 143 (12.1)	9675 (13.9)^b^	10 468 (10.8)	−0.1
Ever any bankruptcy filing submitted (past 90 d)	2786 (1.7)	1266 (1.8)^b^	1520 (1.6)	0.0
Ever any foreclosure (past 90 d)	673 (0.4)	341 (0.5)^b^	332 (0.3)	0.0
**Credit score and balances across the study period, mean (SD)**				
No. of adverse financial outcomes (0-7)	1.5 (1.6)	1.7 (1.6)^b^	1.3 (1.6)	−0.2
Lowest Vantage credit score, 300-850 (as of end of quarter continuous)	647 (120)	632 (119)^b^	658 (121)	0.2
Maximum balance of medical debt in collections (of those with any), $	2162 (4 884)	2318 (5232)^b^	2003 (4503)	−0.1
Maximum balance of nonmedical debt in collections (of those with any), $	2584 (4143)	2552 (3953)^b^	2614 (4319)	0.0
Total 60-plus–day delinquent debt (of those with any), $	22 364 (47 970)	19 861 (39 532)	24 491 (54 115)	0.1

Abbreviation: SMD, standardized mean difference.

^a^ Coded as a binary measure of ever having had such outcomes during a given quarter (coded as 1 or as 0 if otherwise).

^b^ *P* < .001.

### Exposure to and Count of Adverse Financial Outcomes

**Figure 1.**
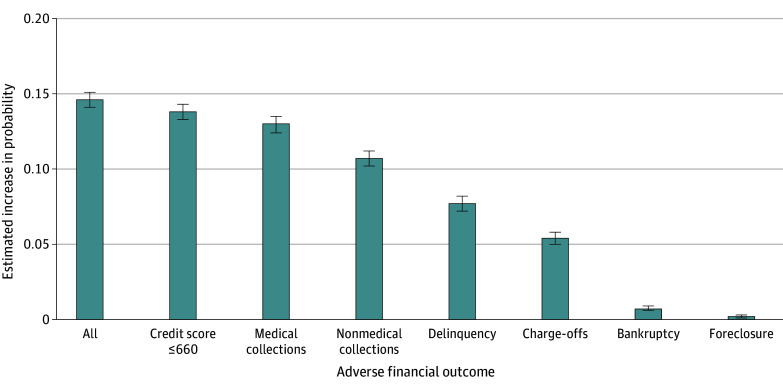
Estimated Increase in the Probability of Having Adverse Financial Outcomes for Patients With and Without Type 2 Diabetes The number of unique patients from October 2017 to December 2021 was 166 285. Results are estimated marginal effects of odds ratios from generalized linear model regression (binomial family) and logistic link function of data. Covariates were patient age, sex, race, Hispanic ethnicity, wage earnings, health insurance type, and calendar-quarter indicators. Error bars indicate SEs, which are clustered on individual patients.

We found greater exposure to all adverse financial outcomes for patients with vs without diabetes (Figure 1; eTable 2 in Supplement 1). In binary logistic regressions, adjusted for sociodemographic characteristics, wage earnings, and insurance type, among patients with vs without diabetes, estimated probabilities were higher for having any adverse financial outcome (64.5% [95% CI, 64.1%-64.9%] vs 49.9% [95% CI, 49.6%-50.2%]), below-prime credit score (59.7% [95% CI, 59.3%-60.1%] vs 45.9% [95% CI, 45.6%-46.2%]), medical collections (36.9% [95% CI, 36.5%-37.3%] vs 23.9% [95% CI, 23.7%-24.2%]), nonmedical collections (38.4% [95% CI, 38.0%-38.8%] vs 27.7% [95% CI, 27.5%-28.0%]), delinquent debt (23.3% [95% CI, 22.9%-23.7%] vs 15.6% [95% CI, 15.4%-15.8%]), debt charge-offs (15.4% [95% CI, 15.1%-15.8%] vs 10.1% [95% CI, 9.9%-10.2%]), bankruptcy filings (2.1% [95% CI, 2.0%-2.3%] vs 1.4% [95% CI, 1.3%-1.5%]), and foreclosures (0.5% [95% CI, 0.5%-0.6%] vs 0.3% [95% CI, 0.2%-0.4%]). All regression estimates for patients with vs without diabetes were significant at *P* < .001.

### Estimated Credit Score and Debt Balances

**Figure 2.**
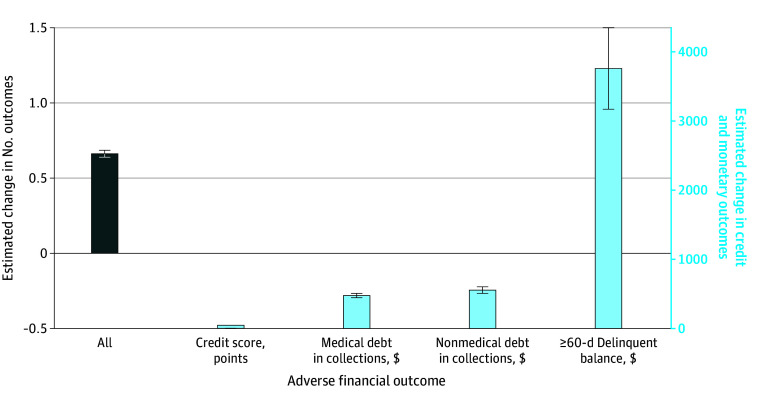
Estimated Change in the Number of Adverse Financial Outcomes, Credit Score, and Dollar Amounts of Medical and Nonmedical Debt in Collections and Delinquent Debt for Patients With and Without Type 2 Diabetes Shown are outcomes of type 2 diabetes for count of adverse financial outcomes (left y-axis) and credit scores, balances of medical and nonmedical debt in collection, and delinquent debt (right y-axis). Results are estimated coefficients from negative binomial regression (adverse financial outcomes), generalized linear models with γ-distributed response and log link (credit score), and 2-part exponential hurdle models (medical and nonmedical debt in collection, delinquent debt). Covariates in all models were patient age, sex, race, Hispanic ethnicity, wage earnings, health insurance types, and calendar-quarter indicators. Error bars indicate SEs, which are clustered on individual patients.

Patients with diabetes had more adverse financial events (1.9 vs 1.2) and a lower mean (SE) credit score (618.7 [0.4] vs 664.2 [0.5]) than patients without type 2 diabetes (Figure 2). Among patients with a nonzero balance (proportions shown in Table 2), patients with vs without diabetes had higher medical collection ($1151 [95% CI, $1122-$1180] vs $716 [95% CI, $699-$734]), nonmedical collection ($1875 [95% CI, $1834-$1916] vs $1361 [95% CI, $1333-$1389]), and delinquent debt ($11 387 [95% CI, $10 796-$11 977] vs $7630 [95% CI, $7305-$7955]) balances (eTable 2 in Supplement 1). All estimates for diabetes were statistically significant at *P* < .001.

In alternative specifications using a generalized estimating equation repeated-measures approach, all estimates of adverse financial outcomes for patients with vs without diabetes were significantly higher (eTable 3 in Supplement 1). Specifications that controlled for prescription of antidiabetic medications and estimates of adverse financial outcomes for patients with vs without diabetes remained significantly higher for most adverse financial outcomes except bankruptcy filing, foreclosure, and balance of delinquent debt (eTable 4 in Supplement 1).

### Heterogeneity by Age, Sex, Race, Hispanic Ethnicity, Health Insurance, and Wage Earnings

**Figure 3.**
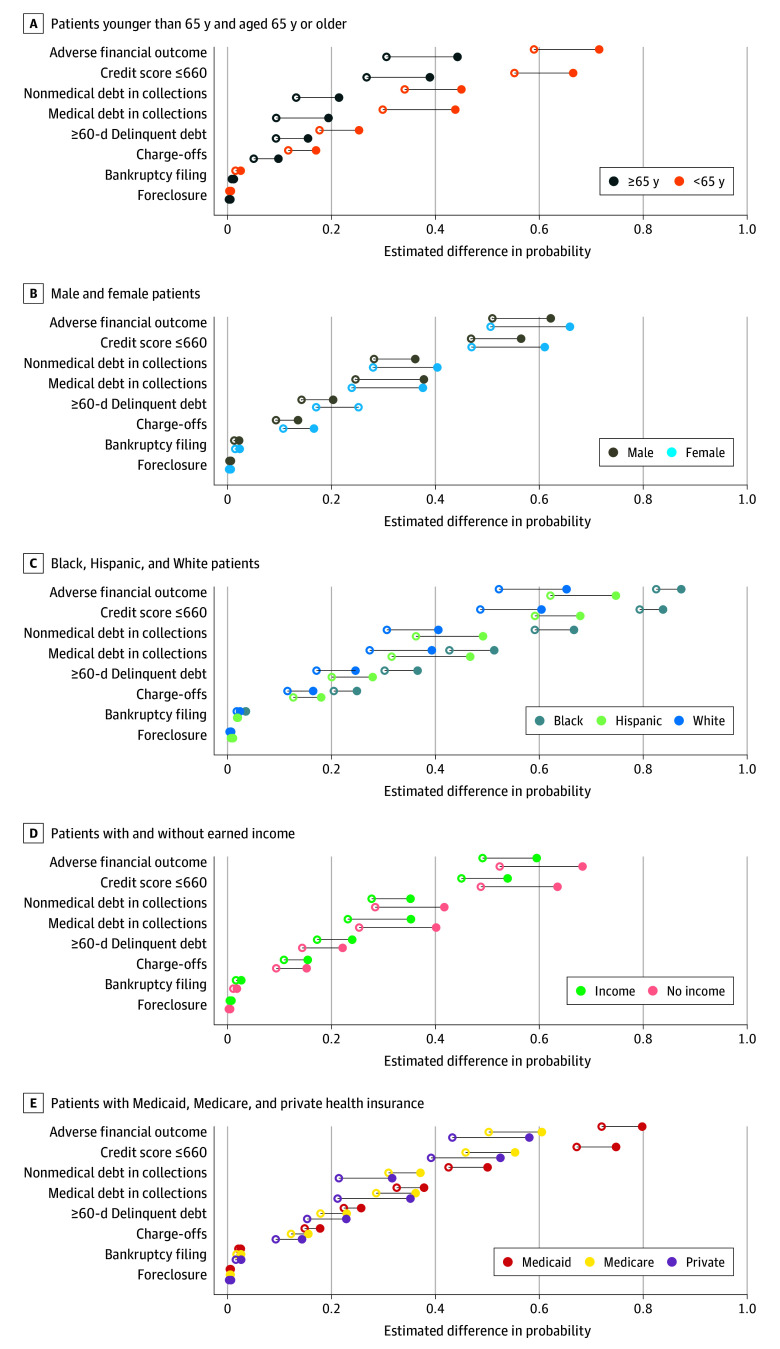
Heterogeneity of Regression Results by Age, Sex, Race, Hispanic Ethnicity, Earned Income, and Insurance Type The solid line indicates the difference between patients with (filled circles) vs without (open circles) type 2 diabetes.

We examined adverse financial outcomes specific to socioeconomic characteristics by interacting the type 2 diabetes indicator with indicators for age, sex, race, Hispanic ethnicity, insurance type, and wage earnings. Estimated adjusted probabilities for all 7 adverse outcomes are shown in Figure 3 and eTable 5 in Supplement 1. Younger patients (aged <65 years) with diabetes were more likely to experience any adverse financial outcomes than older patients with diabetes (71.5% vs 44.3%). However, compared with similarly aged patients without diabetes, the difference in the adjusted probability of any adverse financial outcomes was higher for both older patients (marginal difference, 13.7% [95% CI, 12.6%-14.7%]) and younger patients (marginal difference, 12.5% [95% CI, 11.9%-13.2%]) with diabetes. With regard to sex, female patients with diabetes had higher adjusted probabilities of adverse financial outcomes (marginal effect, 65.9% [95% CI, 65.4%-66.4%]) than male patients with diabetes (marginal effect, 62.2% [95% CI, 61.7%-62.7%]). In addition, the difference in the adjusted probability of adverse financial outcomes was larger for female patients with and without diabetes (marginal difference, 15.3% [95% CI, 14.6%-15.9%]) vs male patients (marginal difference, 11.2% [95% CI, 10.5%-11.9%]).

Black patients with diabetes had higher adjusted probabilities of adverse financial outcomes (marginal effect, 87.3% [95% CI, 86.5%-88.2%]) than White patients with diabetes (59.4% [95% CI, 58.8%-59.9%]), driven by the higher prevalence of adverse financial outcomes among Black patients regardless of diabetes. Compared with patients of the same race without diabetes, the adjusted probability of adverse financial outcomes was only 4.8% (95% CI, 3.6%-6.0%) higher for Black patients with diabetes, while it was 13.0% (95% CI, 12.4%-12.6%) higher for White patients with diabetes. Hispanic patients with diabetes had higher adjusted probabilities of adverse financial outcomes (marginal effect, 74.8% [95% CI, 72.3%-77.2%]) than non-Hispanic patients with diabetes (marginal effect, 64.0% [95% CI, 63.6%-64.4%]). Compared with Hispanic patients without diabetes, the difference in the adjusted probability of adverse financial outcomes was 12.6% (95% CI, 9.6%-15.6%) higher for Hispanic patients with diabetes.

Patients with diabetes without earned income had higher adjusted probabilities of adverse financial outcomes (marginal effect, 68.3% [95% CI, 67.8%-68.9%]) than those with earned income (marginal effect, 59.5% [95% CI, 58.8%-60.2%]). Compared with patients without diabetes with similar earnings situations, the adjusted probability of adverse financial outcomes was higher for patients with diabetes without wage earnings (marginal difference, 15.9% [95% CI, 15.3%-16.6%]) and for those with wage earnings (marginal difference, 10.4% [95% CI, 9.6%-11.2%]). Patients with diabetes ever covered by Medicaid had higher adjusted probabilities of adverse financial outcomes (marginal effect, 79.8% [95% CI, 78.8%-80.8%]) than those ever covered by Medicare (marginal effect, 60.5% [95% CI, 59.8%-61.1%]) or private insurance (marginal effect, 58.1% [95% CI, 57.5%-58.7%]). However, the adjusted probability of adverse financial outcomes between patients with and without diabetes was largest for patients with private insurance (marginal difference, 14.8% [95% CI, 14.1%-15.6%]), followed by Medicare (marginal difference, 10.2% [95% CI, 9.3%-11.2%]), and Medicaid (marginal difference, 7.8% [95% CI, 6.4%-9.2%]). All adjusted probabilities were significant at *P* < .001.

## Discussion

In this economic evaluation of the association of adverse financial outcomes with type 2 diabetes, we found that patients with diabetes at a large Midwestern medical center had significantly higher rates for all 7 examined adverse financial outcomes, including higher rates of below-prime credit scores, medical debt in collections, nonmedical debt in collections, delinquent debt, charge-offs, bankruptcy filings, and foreclosures. The data show that patients with diabetes who had debt in collections and delinquent debt had significantly higher dollar amounts of debt than those without diabetes, as well as significantly lower credit scores. Patients with diabetes were more likely to experience 2 of the 7 financial outcomes compared with 1.2 outcomes of patients without diabetes.

The data point to substantial financial hardships for patients with type 2 diabetes and provide context for rationing behavior,^3^ the lack of adherence to diabetes care recommendations, and lower health care use among these patients.^14^ These findings are important because financial hardships have been associated with mental health problems and higher mortality in the wider financial toxicity literature.^15^

Our study found worse adverse financial outcomes for patients of Black race, enrolled in Medicaid, of Hispanic ethnicity, younger than 65 years, without earned income, and of female sex. Within these groups, the differences in marginal effects between patients with and without diabetes varied. For Black patients or patients with Medicaid insurance, smaller marginal differences represented a higher risk of adverse financial outcomes overall, regardless of a type 2 diabetes diagnosis, that may be associated with ceiling effects or structural inequality. Within other groups, such as older patients and patients with private insurance, the risk of financial hardship was much higher for those with vs without diabetes. These findings highlight the need to consider the intersection of social determinants of health for holistic patient treatment.

The study findings are consistent with prior studies that documented an association of chronic illness with financial hardship. In a study using administrative consumer credit panel data in Washington State from 2013 to 2018, a cancer diagnosis was associated with a lower likelihood of meeting financial obligations (ie, missing credit card payments, debt in collections).^12^ More generally, hospital admission has been associated with significantly higher medical and nonmedical collections, bankruptcy, lower credit scores, and credit limits in California discharge data from 2002 to 2011.^16^ To our knowledge, the current study is among the first to link administrative EHR, credit, and wage earnings data at the individual level to investigate their association with type 2 diabetes. Controlling for health insurance and wage earnings over several years and including an economically diverse sample enabled us to compare outcomes across a wider set of socioeconomic characteristics than in earlier research.^9^

Studies based on survey data of individuals with type 2 diabetes were also consistent with our findings, documenting the association of type 2 diabetes with difficulty paying medical bills, meeting unexpected medical costs, and paying for usual health care.^4^ However, these studies were based on self-reported data, which may be of lower quality because of systematic response biases^17^ and are typically not collected as frequently as quarterly credit record panels.^11,18^

Directions for future research are to examine how financial hardships are associated with the progression of type 2 diabetes and disability-related complications, with a particular focus on heterogeneity by socioeconomic characteristics. This research may inform policymakers about evolving risks to the economic security of financially vulnerable individuals with diabetes. This information also may inform holistic treatment approaches that might ease the financial vulnerability of individuals with diabetes.

### Limitations

This study has several limitations. It is limited to patients at a single Midwestern medical center with encounters between October 2017 and December 2021. This population had a larger share of individuals with type 2 diabetes than the general population (41.7% vs 11.3%). The relative share of demographic groups among patients with type 2 diabetes was also not typical for the US population with type 2 diabetes, for example, in which Hispanic ethnicity accounts for a larger share (10.4% vs 2.0%).^19^ Our comparison group included patients at the medical center without type 2 diabetes indicators but who had an HbA_1c_ test. This comparison group may be systematically different from comparison patients without an HbA_1c_ test, which might systematically bias results and underestimate effect sizes. The income data were limited to wage income. We did not have access to complete personal and household income data for our sample. Other income, such as from financial investments, retirement income, public benefits, and Social Security, were not included in our data. This lack of other income limits conclusions about the role of income, especially for retired adults who rely primarily on retirement and Social Security income. The data also lacked complete information on medication prescriptions, including only medications prescribed at the medical center. Medications prescribed at other health care institutions were not available and may be the reason for lacking prescription information for 19.6% of the patients with type 2 diabetes in the study cohort. The data we could access did not have information on medications filled, overall medication regimen, out-of-pocket costs, or complete comorbidities.

## Conclusions

This economic evaluation study of patients with type 2 diabetes provides novel findings of the multiplicity of financial burdens in these individuals. It documents the prevalence of financial toxicity for type 2 diabetes beyond the well-documented prevalence among patients with cancer.^20,21^ Future research should examine the impact of adverse financial outcomes on type 2 diabetes progression and related complications.

## Notes

Supplement 1. **eFigure.** Flowchart of Analytic Sample Construction **eMethods.** Details About Variable Coding **eTable 1.** Descriptive Statistics of Analytic Sample of The Ohio State University Wexner Medical Center vs 1% Ohio Random Sample of Experian Credit Reports for a Selected Quarter (as of December 31, 2019) **eTable 2.** Summary of Regression Results **eTable 3.** Odds Ratios of Generalized Estimating Equation Regression of Adverse Financial Outcomes on T2D **eTable 4.** Robustness: Odds Ratios of Cross-Sectional Regression of Adverse Financial Outcomes on T2D, Controlling for T2D Medication Prescriptions **eTable 5.** Marginal Effects, Marginal Differences, and Odds Ratios of Interaction Terms of T2D With Socioeconomic Groups for Exposure Variable Ever Any Adverse Financial Outcomes (Figure 3)

Supplement 2.
Data Sharing Statement
