# Supplementary material for: Type 2 Diabetes and Financial Outcomes
Source: JAMA Netw Open. 2025 Jul 28;8(7):e2523453. doi: 10.1001/jamanetworkopen.2025.23453 (PMC12305386; doi:10.1001/jamanetworkopen.2025.23453)
Supplement: Supplement 1. — eFigure. Flowchart of Analytic Sample Construction eMethods. Details About Variable Coding eTable 1. Descriptive Statistics of Analytic Sample of The Ohio State University Wexner Medical Center vs 1% Ohio Random Sample of Experian Credit Reports for a Selected Quarter (as of December 31, 2019) eTable 2. Summary of Regression Results eTable 3. Odds Ratios of Generalized Estimating Equation Regression of Adverse Financial Outcomes on T2D eTable 4. Robustness: Odds Ratios of Cross-Sectional Regression of Adverse Financial Outcomes on T2D, Controlling for T2D Medication Prescriptions eTable 5. Marginal Effects, Marginal Differences, and Odds Ratios of Interaction Terms of T2D With Socioeconomic Groups for Exposure Variable Ever Any Adverse Financial Outcomes (Figure 3) [file jamanetwopen-e2523453-s001.pdf]

Supplemental Online Content

Pesavento M, Loibl C, Moulton S, et al. Financial outcomes and type 2 diabetes. *JAMA Netw Open*. 2025;8(7):e2523453. doi:10.1001/jamanetworkopen.2025.23453

eFigure. Flowchart of Analytic Sample Construction

eMethods. Details About Variable Coding

eTable 1. Descriptive Statistics of Analytic Sample of The Ohio State University Wexner Medical Center vs 1% Ohio Random Sample of Experian Credit Reports for a Selected Quarter (as of December 31, 2019)

eTable 2. Summary of Regression Results

eTable 3. Odds Ratios of Generalized Estimating Equation Regression of Adverse Financial Outcomes on T2D

eTable 4. Robustness: Odds Ratios of Cross-Sectional Regression of Adverse Financial Outcomes on T2D, Controlling for T2D Medication Prescriptions

eTable 5. Marginal Effects, Marginal Differences, and Odds Ratios of Interaction Terms of T2D With Socioeconomic Groups for Exposure Variable Ever Any Adverse Financial Outcomes (Figure 3)

This supplemental material has been provided by the authors to give readers additional information about their work.

© 2025 Pesavento M et al. *JAMA Network Open*.

eFigure. Flowchart of Analytic Sample Construction

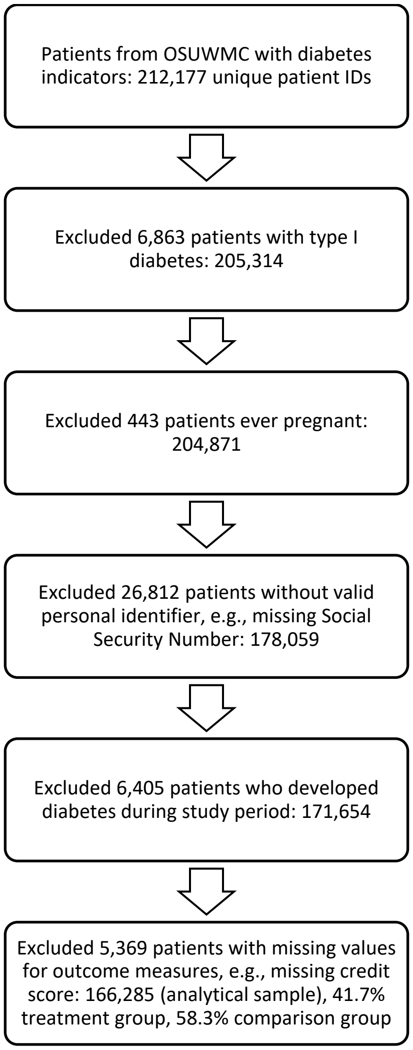

© 2025 Pesavento M et al. *JAMA Network Open*.

eMethods. Details About Variable Coding

VantageScore® Credit Score ≤ 660 (as of end of quarter)
VantageScore® Credit Score, mean (SD), 300-850 (as of end of quarter)
vantage_v4_score

Definitions: *ALL All Trade Types*; *COL Collections*; *MTF First Mortgage*

Any non-medical debt in collections (as of end of quarter)
Balance of non-medical debt in collection (as of end of quarter)

5060 Total balance on collections, COL
5066 Total balance on medical collections, COL
STATA: col5060 – col5066 >= 0 & !=.

Any medical debt in collections (as of end of quarter)
Balance of medical debt in collections (as of end of quarter)

5066 Total balance on medical collections, COL

Any 60-plus day delinquent debt; not derogatory (as of end of quarter)
Total delinquent debt (as of end of quarter)

5043 Total balance on trades presently 60 days delinquent reported in the last 6 Months, ALL
5047 Total balance on trades presently 90-180 days delinquent reported in the last 6 months, ALL
STATA: all5043 >=0 & != . | all5047>=0 & != .

Any charge-offs (past 90 days)

8164 Number of months since the most recent present charge-off including indeterminates, ALL
STATA: all8164 >=0 & <3

Any bankruptcy filing submitted (past 90 days)

9220 Number of months since the most recent public record bankruptcy filed, ALL
STATA: all9220 < 3 & all9220 !=. & all9220 >= 0

Any foreclosure (past 90 days)

8166 Number of months since the most recent presently foreclosed first mortgage trades including indeterminates, MTF
STATA: MTF8166 < 3 & MTF8166 !=. & MTF8166 >= 0

Age

EHR data provided age as of the date of the data draw on March 24, 2022. We backwards extrapolate to 2017 by calculating patients' hypothetical birth year and then imputing patient age in 2017.

```
bysort PatientID (archive): egen approx_yob = min(floor(archive/100)-age)
bysort PatientID (archive): egen age17 = max(floor(2017-approx_yob))
```

© 2025 Pesavento M et al. *JAMA Network Open*.

```
gen age17_034 = inrange(age17,0,34)
gen age17_3549 = inrange(age17,35,49)
gen age17_5064 = inrange(age17,50,64)
gen age17_65pl = age17 >= 65
```

Health Insurance Types

Private Insurance: Patient is covered by employer-based, non-public insurance
Medicare: Patient is covered by Medicare health insurance program
Medicaid: Patient is covered by Medicaid program
Other Insurance: Patients is covered by Bureau of Workers Compensation, Veterans Administration, Bureau of Disability, Columbus Free Clinic, Drug Advocacy Program & Victims of Crime, and other payors
Self pay: Patient does not use insurance to pay for health care costs

© 2025 Pesavento M et al. *JAMA Network Open*.

eTable 1. Descriptive Statistics of Analytic Sample of The Ohio State University Wexner Medical Center vs 1% Ohio Random Sample of Experian Credit Reports (as of December 31, 2019)

	Analytical sample % or Mean SD)	1% Ohio Random Sample % or Mean SD)
Age (mean)	53.62*** (16.4)	49.5 (19.6)
18-24	3.4%*** (0.2)	11.4% (0.3)
25-34	12.1%*** (0.3)	17.1% (0.4)
35-44	15.5%*** (0.4)	14.4% (0.4)
45-54	18.5%*** (0.4)	15.9% (0.4)
55-64	22.8%*** (0.4)	16.8% (0.4)
65-74	17.9%*** (0.4)	12.9% (0.3)
≥75	9.9%*** (0.3)	11.6% (0.3)
Sex		
Male	44.1%*** (0.5)	49.7% (0.5)
Female	55.9%*** (0.5)	50.3% (0.5)
<u>Credit outcomes:</u>		
Vantage Credit Score ≤ 660 (as of end of quarter)	39.5%*** (0.5)	36.3% (0.5)
Any non-medical debt in collections (as of end of quarter)	21.8%*** (0.4)	17.7% (0.4)
Any medical debt in collections (as of end of quarter)	18.2%*** (0.4)	15.5% (0.4)
Any 60-plus day delinquent debt (as of end of quarter)	4.6%* (0.2)	4.8% (0.2)
Any charge-offs (past 90 days)	1.8% (0.1)	1.7% (0.1)
Any bankruptcy filing submitted (past 90 days)	0.1%* (0.04)	0.1% (0.03)
Any foreclosure (past 90 days)	0.1% (0.03)	0.1% (0.03)
Any adverse financial outcome (past 90 days)	42.1%*** (0.5)	39.8% (0.5)
<u>Count credit score and balances:</u>		
No. of adverse financial outcomes	0.84*** (1.1)	0.75 (1.1)
Vantage Credit Score, mean (SD), 300-850 points (as of end of quarter)	692*** (115)	696 (109)
Balance of non-medical debt in collection ($0 included)	$439*** ($1,700)	$350 ($1,478)
Balance of medical debt in collections ($0 included)	$291*** ($1,670)	$210 ($1,332)
Total delinquent debt ($0 included)	$964 ($11,363)	$912 ($9,957)
N (Q4 2019)	146,736	88,176

Notes: * P<0.05, ** P<0.01, *** P<0.001

1% Ohio Random Sample is an approximate 1% random sample of credit records of Ohioans in Quarter 4 of 2019. The credit panel does not include race and ethnicity information

Compared to the 1% random sample of the adult population in Ohio with credit data as of December 2019, the study sample was older (aged 54 vs. 50), more often female (56% vs. 50%), and were more likely to have an adverse financial outcome (42.1% vs. 39.8%).

© 2025 Pesavento M et al. *JAMA Network Open*.

eTable 2. Summary of Regression Results

	T2D (1=yes)<br>OR (95% CI)	Marginal Difference<br>% (95% CI)	Patient with T2D (Margin)<br>% (95% CI)	Patient without T2D (Margin)<br>% (95% CI)	Mean of DV
Credit outcomes:					
Ever any adverse financial outcomes	2.26***<br>(2.19-2.33)	14.6%<br>(14.1%-15.1%)	64.5%<br>(64.1%-64.9%)	49.9%<br>(49.6%-50.2%)	0.56
Ever Vantage Credit Score ≤ 660	2.17***<br>(2.11-2.24)	13.8%<br>(13.3%-14.3%)	59.7%<br>(59.3%-60.1%)	45.9%<br>(45.6%-46.2%)	0.52
Ever any medical debt in collections	2.03***<br>(1.97-2.09)	13.0%<br>(12.4-13.5%)	36.9%<br>(36.5%-37.3%)	23.9%<br>(23.7%-24.2%)	0.29
Ever any non-medical debt in collections	1.93***<br>(1.87-1.99)	10.7%<br>(10.2%-11.2%)	38.4%<br>(38.0%-38.8%)	27.7%<br>(27.5%-28.0%)	0.32
Ever any 60-plus day delinquent debt	1.74***<br>(1.68-1.80)	7.7%<br>(7.2%-8.2%)	23.3%<br>(22.9%-23.7%)	15.6%<br>(15.4%-15.8%)	0.19
Ever any debt charge-off	1.70***<br>(1.64-1.77)	5.4%<br>(5.0; 5.8%)	15.4%<br>(15.1%-15.8%)	10.1%<br>(9.9%-10.2%)	0.12
Every any bankruptcy filing	1.53***<br>(1.40-1.68)	0.7%<br>(0.6%-0.9%)	2.1%<br>(2.0%-2.3%)	1.4%<br>(1.3%-1.5%)	0.02
Ever any foreclosure	1.63***<br>(1.35-1.96)	0.2%<br>(0.1%-0.3%)	0.5%<br>(0.5%-0.6%)	0.3%<br>(0.2%-0.4%)	0.004
Count, credit score and balances:					
Count of adverse financial outcomes	1.54***<br>(1.52-1.56)	0.7<br>(0.64-0.69)	1.9<br>(1.9-1.9)	1.2<br>(1.2-1.2)	1.46
Minimum Vantage Credit Score, 300 to 850 points	1.25***<br>(1.24-1.25)	-46<br>(44-47)	619<br>(618-620)	664<br>(663-665)	647
Maximum balance of medical debt in collections (of those with this debt)	1.13***<br>(1.10-1.17)	$435<br>($403-$467)	$1,151<br>($1,122-$1,180)	$716<br>($699-$734)	$632
Maximum balance of non-medical debt in collection (of those with this debt)	1.11***<br>(1.08-1.14)	$514<br>($466-$563)	$1,875<br>($1,834-$1,916)	$1,361<br>($1,333-$1,389)	$832
Maximum 60-plus day delinquent debt (of those with this debt)	1.05<br>(0.99-1.11)	$3,757<br>($3,160; $4,353)	$11,387<br>($10,796-$11,977)	$7,630<br>($7,305-$7,955)	$4,135

Notes: * P<0.05, ** P<0.01, *** P<0.001; n=166,285
Controlled for Age (Ref.: 50-64): 18-34, 35-49, 65 and older; Female; Race (Ref.: White): Black, Asian, Native, Multiple races, Race missing; Hispanic Ethnicity; Wage Earnings (Ref.: $10,000-$19,999): $0; $1-$9,999, $20,000-$29,999, $30,000-$39,999, $40,000 and more; Interaction $0*age 65 plus; Private Insurance; Medicare; Medicaid; Other Insurance; Self pay; Number quarter in sample; 17 Quarter-year indicators (Q4 2017 to Q4 2021; Ref.: Q4 2021)

© 2025 Pesavento M et al. *JAMA Network Open*.

eTable 3. Odds Ratios of Generalized Estimating Equation Regression of Adverse Financial Outcomes on Type 2 Diabetes

	T2D	Marginal Difference	Patient with T2D (Margin)	Patient without T2D (Margin)	Mean of DV
	OR (95% CI)	% (95% CI)	% (95% CI)	% (95% CI)	
Credit outcomes:					
Any adverse financial outcomes	2.09*** (2.04-2.13)	16.1% (15.6%-16.5%)	51.1% (50.8%-51.4%)	35.1% (34.8%-35.3%)	0.41
Vantage Credit Score ≤ 660	1.95*** (1.91-1.99)	14.2% (13.8%-14.6%)	47.7% (47.4%-48.0%)	33.5% (33.3%-33.8%)	0.38
Any medical debt in collections	1.96*** (1.91-2.01)	10.0% (9.6%-10.4%)	24.2% (23.9%-24.5%)	14.2% (14.0%-14.4%)	0.18
Any non-medical debt in collections	1.78*** (1.74-1.82)	9.1% (8.7%-9.4%)	27.2% (26.9%-27.5%)	18.1% (17.9%-18.3%)	0.21
Any 60-plus day delinquent debt	1.54*** (1.49-1.58)	1.6% (1.5%-1.7%)	4.7% (4.7%-4.8%)	3.2% (3.1%-3.2%)	0.04
Any debt charge-off	1.59*** (1.53-1.65)	0.7% (0.7%-0.8%)	2.0% (2.0%-2.1%)	1.3% (1.3%-1.3%)	0.02
Any bankruptcy filing	1.50*** (1.38-1.63)	0.1% (0.0%-0.1%)	0.2% (0.1%-0.2%)	0.1% (0.1%-0.1%)	0.001
Count and credit score:					
Count of adverse financial outcomes	1.54*** (1.52-1.56)	0.36 (0.35-0.37)	1.0 (1.0-1.0)	0.7 (0.7-0.7)	0.81
Vantage Credit Score, 300 to 850 points	1.26*** (1.25-1.26)	36 (35-38)	671 (670-672)	707 (706-708)	154

Notes: * P<0.05, ** P<0.01, *** P<0.001; n=166,285 patients, n observations=2,412,004; average observations by patient: 14.5 (1 to 17)
Quarterly panel data of 17 time points, from Q4 2017 to Q4 2021. The data are analyzed using generalized estimating equations (GEE) population-averaged panel models. Controlled for Age (Ref.: 50-64): 18-34, 35-49, 65 and older; Female; Race (Ref.: White): Black, Asian, Native, Multiple races, Race missing; Hispanic Ethnicity; Wage Earnings (Ref.: $10,000-$19,999); $0; $1-$9,999; $20,000-$29,999; $30,000-$39,999; $40,000 and more; Interaction $0*age 65 plus; 17 Quarter-year indicators (Q4 2017 to Q4 2021; Ref.: Q4 2017)

© 2025 Pesavento M et al. *JAMA Network Open*.

eTable 4. Robustness: Odds Ratios of Cross-Sectional Regression of Adverse Financial Outcomes on T2D, Controlling for T2D Medication Prescriptions

	T2D (yes=1)<br>OR<br>(95% CI)	Marginal Difference<br>% (95% CI)	T2D medication prescription<br>OR<br>(95% CI)	Insulin prescription<br>OR<br>(95% CI)	Unknown prescription<br>OR<br>(95% CI)	Mean of DV
Credit outcomes:						
Ever any adverse financial outcomes	2.00***<br>(1.85-2.16)	12.4%<br>(11.1%-13.8%)	0.91**<br>(0.85-0.97)	1.56***<br>(1.49-1.63)	1.06<br>(0.98-1.15)	0.56
Ever Vantage Credit Score ≤ 660	1.95***<br>(1.81-2.10)	11.9%<br>(10.5%-13.2%)	0.90**<br>(0.84-0.96)	1.52***<br>(1.46-1.59)	1.07<br>(0.99-1.16)	0.52
Ever any medical debt in collections	2.12***<br>(1.97-2.27)	13.7%<br>(12.4%-15.0%)	0.80***<br>(0.76-0.85)	1.37***<br>(1.31-1.42)	0.92* (0.86-1.00)	0.29
Ever any non-medical debt in collections	1.74***<br>(1.62-1.88)	9.0%<br>(7.8%-10.2)	0.93*<br>(0.87-0.99)	1.40***<br>(1.34-1.46)	1.07<br>(0.99-1.16)	0.32
Ever any 60-plus day delinquent debt	1.59***<br>(1.46-1.73)	6.4%<br>(5.2%-7.6%)	1.00<br>(0.93-1.07)	1.25***<br>(1.19-1.31)	1.00<br>(0.91-1.09)	0.19
Ever any debt charge-off	1.56***<br>(1.41-1.72)	4.5%<br>(3.5%-5.5%)	0.98<br>(0.90-1.07)	1.25***<br>(1.18-1.32)	1.03<br>(0.93-1.14)	0.12
Every any bankruptcy filing	1.05<br>(0.80-1.38)	0.1%<br>(-0.4%-0.5%)	1.48**<br>(1.15-1.90)	1.08<br>(0.95-1.23)	1.27<br>(0.95-1.72)	0.02
Ever any foreclosure	1.245<br>(0.80-1.93)	0.1%<br>(-0.1%-0.3%)	1.11<br>(0.77-1.61)	1.49**<br>(1.16-1.91)	1.02<br>(0.62-1.67)	0.004
Count, credit score and balances:						
Count of adverse financial outcomes	1.49***<br>(1.44-1.54)	0.61<br>(0.56-0.66)	0.94***<br>(0.91-0.96)	1.19***<br>(1.17-1.22)	1.00<br>(0.97-1.04)	1.46
Minimum Vantage Credit Score, 300 to 850 points	1.23***<br>(1.21-1.25)	42<br>(39-46)	0.96***<br>(0.95-0.98)	1.10***<br>(1.09-1.11)	1.00<br>(0.98-1.02);	202
Maximum balance of medical debt in collections (of those with this debt)	1.20***<br>(1.12-1.28)	$510<br>($436-$584)	0.87***<br>(0.82-0.92)	1.11***<br>(1.07-1.16)	0.96<br>(0.89-1.03)	$632
Maximum balance of non-medical debt in collection (of those with this debt)	1.14***<br>(1.07-1.22)	$510<br>($397-$622)	0.96<br>(0.91-1.01)	1.02<br>(0.99-1.06)	0.96<br>(0.89-1.02)	$832
Maximum 60-plus day delinquent debt (of those with this debt)	1.00<br>(0.88-1.14)	$2,730<br>($1,370-$4,090)	1.04<br>(0.93-1.16)	1.01<br>(0.94-1.09)	1.06<br>(0.92-1.22)	$4,135

Notes: * P<0.05, ** P<0.01, *** P<0.001; n=166,285
Controlled for Age (Ref.: 50-64): 18-34, 35-49, 65 and older; Female; Race (Ref.: White): Black, Asian, Native, Multiple races, Race missing; Hispanic Ethnicity; Wage Earnings (Ref.: $10,000-$19,999): $0; $1-$9,999, $20,000-$29,999, $30,000-$39,999, $40,000 and more; Interaction $0*age 65 plus; Private Insurance; Medicare; Medicaid; Other Insurance; Self pay; Number quarter in sample; 17 Quarter-year indicators (Q4 2017 to Q4 2021; Ref.: Q4 2021)
Prescriptions among those with T2D (ever in study period): T2D Medication 48,697 (70.2%); Insulin & Sensitizing Agents 28,569 (41.2%); Unknown T2D prescription 13 624 (19.6%)

© 2025 Pesavento M et al. *JAMA Network Open*.

eTable 5. Marginal Effects, Marginal Differences, and Odds Ratios of Interaction Terms of T2D With Socioeconomic Groups for Exposure Variable Ever Any Adverse Financial Outcomes (Figure 3)

	Marginal Effect % (95% CI)	Marginal Difference % (95% CI)	Interaction term OR (S.E.)
Panel A: Age			
T2D*Under age 65 (=0)	71.5*** (71.1-72.0)		
T2D*65 year plus (=1)	44.3*** (43.5-45.0)		1.18*** (0.05)
65 year plus: T2D vs no T2D		13.7*** (12.6-14.7)	
Under age 65: T2D vs no T2D		12.5*** (11.9-13.2)	
Panel B: Sex			
T2D*Male (=0)	62.2*** (61.7-62.7)		
T2D*Female (=1)	65.9*** (65.4-66.4)		1.25*** (0.03)
Male: T2D vs no T2D		11.2*** (10.5-11.9)	
Female: T2D vs no T2D		15.3*** (14.6-15.9)	
Panel C: Race and Ethnicity			
T2D*Not Black (=0; not shown)	59.4*** (58.8-59.9)		
T2D*Black (=1)	87.3*** (86.5-88.2)		0.70*** (0.04)
T2D*Not White (=0; not shown)	58.8*** (57.5-60.1)		
T2D*White (=1)	65.2*** (64.8-65.7)		0.93 n.s. (0.05)
T2D*Not Hispanic (=0; not shown)	64.0*** (63.6-64.4)		
T2D*Hispanic (=1)	74.8*** (72.3-77.2)		0.99 n.s. (0.10)
Black: T2D vs no T2D		4.8*** (3.6-6.0)	
White: T2D vs no T2D		13.0*** (12.4-12.6)	
Hispanic: T2D vs no T2D		12.6*** (9.6-15.6)	
Panel D: Earned Income			
T2D*Has earned income (=0)	59.5*** (58.8-60.2)		
T2D*No earned income (=1)	68.3*** (67.8-68.9)		1.36*** (0.04)
Has earned income: T2D vs no T2D		10.4*** (9.6-11.2)	
No earned income: T2D vs no T2D		15.9*** (15.3-16.6)	
Panel E: Health Insurance			
T2D * Never Medicaid (=0, not shown)	62.3*** (61.9-62.8)		
T2D * Medicaid (=1)	79.8*** (78.8-80.8)		0.77*** (0.04)
T2D * Never Medicare (=0, not shown)	66.3*** (65.7-66.8)		
T2D * Medicare (=1)	60.5*** (59.8-61.1)		0.76*** (0.03)
T2D * Never Private Insurance (=0, not shown)	71.6*** (71.1-72.1)		
T2D * Private insurance (=1)	58.1*** (57.5-58.7)		1.26*** (0.05)
Medicare: T2D vs no T2D		7.8*** (6.4-9.2)	
Medicaid: T2D vs no T2D		10.2*** (9.3-11.2)	
Private insurance: T2D vs no T2D		14.8*** (14.1-15.6)	

Notes: * P<0.05, ** P<0.01, *** P<0.001; n=166,285; Results of the remaining interaction terms shown in Figure 3 are available upon request.

Variables in this specification: T2D, Age (<65, ≥65); Female; White, Black, Hispanic Ethnicity; Wage Earnings (Ref.: $10,000-$19,999): $0; $1-$9,999, $20,000-$29,999, $30,000-$39,999, $40,000 and more; Private Insurance; Medicare; Medicaid; Number quarter in sample; 17 Quarter-year indicators (Q4 2017 to Q4 2021; Ref.: Q4 2021) plus T2D*Age 65 plus; T2D*Black; T2D*White; T2D*Hispanic; T2D*$0 Wage Earnings; T2D*Female; T2D*Medicaid; T2D*Medicare; T2D*Private insurance

© 2025 Pesavento M et al. *JAMA Network Open*.
