# Supplementary material for: Type 2 Diabetes and Financial Outcomes
Source: JAMA Netw Open. 2025 Jul 28;8(7):e2523453. doi: 10.1001/jamanetworkopen.2025.23453 (PMC12305386; doi:10.1001/jamanetworkopen.2025.23453)
Supplement: Supplement 2. — Data Sharing Statement [file jamanetwopen-e2523453-s002.pdf]

Data Sharing Statement

Data
Data available: No

Additional Information
Explanation for why data not available: The study data are subject to data use agreements which prohibit our making these data publicly available.
